# Case-controlled Analysis of Patient-based Risk Factors for Assault in the Healthcare Workplace

**DOI:** 10.5811/westjem.2017.7.34845

**Published:** 2017-09-18

**Authors:** Ilene A. Claudius, Shoma Desai, Ebony Davis, Sean Henderson

**Affiliations:** University of Southern California, Keck School of Medicine, Department of Emergency Medicine, Los Angeles, California

## Abstract

**Introduction:**

Violence against healthcare workers in the medical setting is common and associated with both physical and psychological adversity. The objective of this study was to identify features associated with assailants to allow early identification of patients at risk for committing an assault in the healthcare setting.

**Methods:**

We used the hospital database for reporting assaults to identify cases from July 2011 through June 2013. Medical records were reviewed for the assailant’s (patient’s) past medical and social history, primary medical complaints, ED diagnoses, medications prescribed, presence of an involuntary psychiatric hold, prior assaultive behavior, history of reported illicit drug use, and frequency of visits to same hospital requesting prescription for pain medications. We selected matched controls at random for comparison. The primary outcome measure(s) reported are features of patients committing an assault while undergoing medical or psychiatric treatment within the medical center.

**Results:**

We identified 92 novel visits associated with an assault. History of an involuntary psychiatric hold was noted in 52%, history of psychosis in 49%, a history of violence in the ED on a prior visit in 45%, aggression at index visit noted in the ED chart in 64%, an involuntary hold (or consideration of) for danger to others in 61%, repeat visits for pain medication in 9%, and history of illicit drug use in 33%. Compared with matched controls, all these factors were significantly different.

**Conclusion:**

Patients with obvious risk factors for assault, such as history of assault, psychosis, and involuntary psychiatric holds, have a substantially greater chance of committing an assault in the healthcare setting. These risk factors can easily be identified and greater security attention given to the patient.

## INTRODUCTION

Violence directed at healthcare workers (HCW) is not uncommon. In the U.S., 13% of healthcare employees have reported at least one assault,^1^ and 1.9 physical assaults resulting in an injury occur for every 100,000 worker hours.^2^ The incidence of HCW assault in the U.S. is 1.65^3^ or a median of 11 physical attacks per year per site.^4^ Physical assaults comprise 6–21%^5^ of all threatening behavior to which HCW are exposed, with verbal assaults, threats, and property damage accounting for the balance.^6^,^7^ Clearly, this is not evenly distributed throughout hospitals and provider type. Psychiatric, rehabilitation, and geriatric areas have all been shown to have a higher number of assaults.^8^,^9^ As the emergency department (ED) is the front line for these patients, often in their most decompensated state, the threat of aggression toward HCW is a significant concern for ED providers.^10^ In a survey study, 78% of emergency medicine residents or attending physicians reported being the victim of at least one act of workplace violence.^5^

Many reports of risk factors in the healthcare workplace have focused on the HCW themselves. Females, individuals older than 50 years of age, and staff members with longer tenure reported higher numbers of assaults and of fear.^4^,^11^ Patient-staff conflicts substantially correlated and intra-staff conflict moderately related to frequency of assault.^12^ The psychiatric literature has identified a history of violent episodes,^13^,^14^ psychosis,^14^ drug misuse, 14 paranoid schizophrenia,^15^ and anti-social personality disorder 16,17 as predictors of violence. However, these associations are primarily studied in inpatient psychiatric facilities or general criminal behavior. Predictors in the emergency and acute healthcare setting are lacking. One patient-oriented cause that has been identified is dissatisfaction with care.^18^ Unfortunately, often this is not shared with the healthcare team until the violence has occurred. Others have been suggested by interviews of ED personnel, including psychiatric patients, anxiety, staring, mumbling, pacing, and gang violence.^19^,^20^,^21^,^22^ The goal of this study was to use data from reported HCW assaults to begin to identify more clearly patient-level risk factors associated with assaultive behavior in the ED or following admission through the ED.

## METHODS

### Setting

LAC+USC is a large, urban, county hospital with an ED census of 180,000 visits per year. There is a psychiatric ED and an affiliated off-premises inpatient psychiatric facility. In-hospital assaults by patients are reported through a standardized process. Assaults can be reported when they occur anywhere on the hospital grounds, including the medical floor, the outlying affiliated psychiatric facility, the ED, the outpatient clinics, and guest areas.

### Patients

We examined all reports of assaults committed by patients from July 2011 through June 2013. We excluded cases if the assailant could not be identified by the report or if there was incomplete information on the assailant in the medical record. If a single patient committed multiple assaults during the same hospital stay, only the first assault was included. For each unique patient, an attempt was made to include two age- and gender-matched controls with an ED visit within three days of the patient. We reviewed medical records of the controls, including a search for any available psychiatric consultations or notes.

### Study Design

This was a retrospective case-control study design. We collected cases from the hospital assault database. Once the cases were identified, hospital medical records were acquired and reviewed for further information about the assailant. For each assault, we recorded the assailant’s (patient’s) past medical and social history, primary medical complaints, ED diagnoses, and medications prescribed. Additionally, presence of an involuntary psychiatric hold and involuntary restraints by either physical or chemical means during the index visit were recorded. We also reviewed prior records for prior assaultive behavior, history of reported illicit drug use, and frequency of visits to same hospital requesting prescription for pain medications. There were three assessors: the study principal investigator (PI), a second ED attending physician, and a student. Both the attending physician and the student received training and oversight by the PI. About 10% of the assault cases were coded by both, and the agreement was assessed. The assessors were not blinded to the study intent. This study was approved by the University of Southern California, institutional review board.

**Population Health Research Capsule**

What do we already know about this issue?

Assaults in the healthcare setting impact 13% of employees and impact job satisfaction, work attendance and patient outcomes.

What was the research question?

Could patient-level risk factors for in-hospital violence be identified?

What was the major finding of the study?

Most in-hospital assailants had a current or past involuntary psychiatric hold, psychosis, or aggression.

How does this improve population health?

Improving safety in the ED and the hospital is beneficial to healthcare workers, as well as to other potential victims, such as visitors and other patients.

### Definitions

We classified any documented evidence of verbal or physical aggression witnessed by staff or reported by observers as aggressive behavior. If occurring in the ED, this was labeled ED aggression and included verbal threats, name-calling toward staff, true physical assaults, and physical gestures interpreted as threatening (e.g. purposefully swinging IV pole toward provider). In California, an involuntary hold is titled 5150 (or 5585 for children). If this was cited in the chart or ordered, we considered the patient to be on an involuntary hold. The hold itself was then referenced for cause. If the patient had an involuntary hold listed under past medical history in the notes from their reference visit, listed as a diagnosis on the computerized list of medical problems, or cited in a note from a prior visit, then the patient was considered to have a past medical history of a psychiatric complaint.

Illicit drug use was considered positive if patient report of drug use or a positive urine toxicology screen was documented. We did not include marijuana as an illicit drug, as marijuana for medical reasons was legalized in California during the time of data collection. We considered five or more visits requesting opioid pain medication in the year prior to the assault visit as repeat visits for pain medication.

### Statistical Analysis

We assessed differences between case and control population by unadjusted odds ratios (OR) using logistic regressions. Statistical analyses were performed using Stata 13 (StataCorp, 2013) using two-tailed tests with α set to 0.05.

## RESULTS

In total, we identified 117 assaults by patients. Inadequate documentation resulted in exclusion of 10 patients. We excluded 15 additional records due to multiple assaults by the same patient during the same visit (11 patients had two assaults reported and two patients had three assaults reported), leaving 92 assaults for analysis. The majority of the attacks (93.5%) included a physical component; the remainder were verbal. Males were slightly over-represented (59%), and the assailant age range was 9 – 78 years (mean 39 years). Seven of the patients used medical equipment as a weapon, including charts, pencils, tape, linen hampers, and boxes of gloves. Seven physicians, 18 nursing assistants (NA), and 29 registered nurses were involved in assaults, with the balance comprised of other staff, food service personnel, unspecified multiple staff members, and visitors.

The most common location of reported assaults (37%) was the inpatient psychiatric ward; however, 27% occurred in the medical inpatient areas, and 31% in the ED. The most common historical features of the assailants were a history of an involuntary psychiatric hold (52%) and a history of psychosis (49%). A history of danger to others was documented in 47% and of danger to self in 48%. Forty-five percent had a history of violence in the ED on a prior visit. During the stay in which the assault occurred, 64% of the patients had aggression noted in the ED chart, and 61% were on or under consideration for an involuntary hold for danger to others. Repeat visits for pain medication use (9%) and history of drug use (33%) were statistically more common among assailants, but did not seem to be major drivers of aggression in the medical environment. Agreement between chart reviewers was 70%.

We identified a total of 179 matched controls with complete records. Of these, one patient had a history of psychosis and 11 (6%) had a history of an involuntary psychiatric hold for any reason. Six (4%) were aggressive in the ED, and five (3%) were under consideration or on an involuntary danger to others hold. For all features studied, except for history of anxiety and depression, there was a significant difference between assaultive patients and matched controls. History of psychosis (OR 170.4), history of involuntary hold for danger to others (OR 51.5), ED aggression noted (OR 50.4), and current consideration for involuntary danger to others hold (OR 52.8) all had ORs >50 for committing a reported assault. This data is summarized in Table 1.

When the patients who first assaulted a HCW on the inpatient wards or inpatient psychiatric facility were analyzed separately, 71% were either psychotic or on an involuntary hold for danger to others in the ED (vs 11% of controls); 60% demonstrated aggressive behavior in the ED (compared with 4% of controls); and 50% required code activation for restraints in the ED (vs 2% of controls) (Table 2).

## DISCUSSION

Violence against HCW is a problem and, when it occurs, can lead to unacceptable outcomes. It is not unusual for emergency personnel to feel that violence is endemic to the workplace and that acceptance of violence is part of the culture of the workplace.^23^ In this study, nearly 94% of attacks reported here were of a physical nature, making it likely that threats and verbal attacks in isolation were under-reported. Violent experiences in the workplace impact workers’ commitment to the facility,^24^ job satisfaction, and patient outcomes.^25^,^26^ Psychological effects can linger for weeks to months following an incident.^27^ Missed work days and legal fees cause financial impact as well. While a few studies have investigated programs to support or train HCW, the problem remains rampant.

Features such as gender and duration of employment of HCW have been identified, but these do not extrapolate to obvious interventions on the level of a physician-patient interaction. In this study, we identified some predictors of HCW assault. The majority of features were not surprising. Patients with a history of psychosis, aggression demonstrated in the ED, or currently or previously on/under consideration of an involuntary hold for danger to others were logically more likely to assault a HCW. This deviates slightly from prior work on community violence only implicating mental illness as a predictor of violence when co-occurring with substance abuse and/or dependence.^28^

The self-evident nature of these risk factors makes assailant identification and implementation of useful interventions easier. Patients with a known disclosure or behavior indicating interest in harming others, psychosis, and aggressive behavior early in the visit may require a higher level of security and an enhanced level of caution on the part of the healthcare providers. While many patients falling into these categories do not commit an assault, and clearly individual rights cannot be infringed upon needlessly, these patients should be flagged as higher risk of assault and appropriate safety measures enacted to protect both patient and staff from violence.

Many of the victims in this data set were NAs, which is likely because NAs are used as sitters at our facility. NAs or comparable staff with no defense training or clear support plan should not be left alone with the responsibility of sitting for patients at high risk of assaulting a HCW. Targeting certain patients for a higher level of security would have identified the majority of assailants in this study. This pilot study provides some preliminary predictive features to guide in the identification of which patient require heightened security.

## LIMITATIONS

Clearly, documentation of many of the data points was highly dependent on the patient’s primary complaint. Patients seen for psychosis often had an extensive psychiatric history documented; those presenting with medical complaints frequently did not. While prior records were helpful, visits to other hospitals were not assessed. With these limitations, the reported histories of aggression and psychiatric disorders were likely underestimated, although the underestimation was likely greater in the control population.

Additionally, it is likely that not all assaults are reported via this system. Healthcare workers may not feel it necessary or take the time to report more minor or aborted attempts at violence. The nature of self-report tends to select for more significant assaults and may bias the data to include patients with a stronger history of violence and mental health issues. Prior literature cites reporting rates as low as 19%.^29^ The rate of physical assault in this study is much higher than that reported in survey and interview-based literature. Likely the most significant assaults were more frequently reported, while verbally aggressive and threatening patients were overlooked.

## CONCLUSION

Patients with a history of psychosis or of an involuntary psychiatric hold, as well as those expressing aggression in the ED and those on or under consideration of an involuntary psychiatric hold for danger to others should be considered a higher risk group for committing an assault against a healthcare worker. Resources should be dedicated to the observation and intervention of this patient population.

## Figures and Tables

**Table 1:** Assault/assailant characteristics of patients with propensity for aggressive behavior.

Characteristic	N (%)	95% CI	Control % (p-value)	OR
Assault type				
Physical	76 (83%)			
Verbal	6 (7%)			
Both	10 (11%)			
Assault location				
Inpatient psychiatric facility	34 (37%)			
Inpatient medical ward	25 (27%)			
ED	29 (31%)			
Public area	3 (3%)			
Radiology	1 (1%)			
Psychiatric history				
Involuntary hold	48 (52%)	41.8–62.6	6% (<0.001)	16.7
Danger to self	44 (48%)	34.7–58.2	6% (<0.001)	14
Danger to others	43 (47%)	36.3–57.1	2% (<0.001)	51.5
Psychosis	45 (49%)	38.5–59.3	1% (<0.001)	170.4
Schizophrenia	27 (29%)	19.9–38.8	5% (<0.001)	7.8
Bipolar	25 (27%)	17.9–36.4	4% (<0.001)	7.8
Depression	18 (20%)	11.3–27.8	11% (0.05)	2
Anxiety	9 (10%)	3.6–16	4% (0.06)	2.7
None	13 (14%)	7.9–21.4	80% (<0.001)	
History of aggressive behavior				
ED	41 (45%)	34.2–54.9	2% (<0.001)	47.2
Inpatient	19 (21%)	12.2–29.1	2% (<0.001)	11.4
Outpatient	7 (8%)	2.1–13.1	0%	
None	48 (52%)	41.8–62.6	98% (<0.001)	
Social history				
Illicit drug use	30 (33%)	22.8–42.4	12% (0.00)	3.6
>5 visits for pain medication/1y	8 (9%)	2.9–14.7	<1%	
ED course of index visit				
Aggression noted	59 (64%)	54.8–74.8	4% (<0.001)	50.4
Hold for danger to others	56 (61%)	51.4–71.7	3% (<0.001)	52.8
Psychosis	49 (53%)	43.3–64.3	6% (<0.001)	16.9
Code team called for restraint	46 (50%)	40.6–61.6	1% (<0.001)	30.3
Psychiatric medications given	45 (49%)	39–60	5% (<0.001)	19.8

*CI*, confidence interval; *ED*, emergency department; *OR*, odds ratio.

**Table 2:** Assaults occurring on inpatient units.

Characteristic	Assaults (N=59)	Controls (N=114)	p value
Age (years)	38.3	38.3	0.99
Female (%)	49	49	0.99
Psychosis or DTO hold in ED	71%	11%	<0.001
Aggressive behavior in ED	60%	4%	<0.001
Activation for restraints in ED	50%	2%	<0.001

*DTO*, danger to others; *ED*, emergency department.
